# Common Mental Disorders among Occupational Groups: Contributions of the Latent Class Model

**DOI:** 10.1155/2016/3475801

**Published:** 2016-08-17

**Authors:** Kionna Oliveira Bernardes Santos, Fernando Martins Carvalho, Tânia Maria de Araújo

**Affiliations:** ^1^Physical Therapy Department, Federal University of Bahia (UFBA), Avenida Reitor Miguel Calmon s/n, Vale do Canela, 40.110-100 Salvador, BA, Brazil; ^2^Department of Preventive and Social Medicine, Federal University of Bahia (UFBA), Avenida Reitor Miguel Calmon s/n, Vale do Canela, 40.110-100 Salvador, BA, Brazil; ^3^Health Department, State University of Feira de Santana (UEFS), Avenida Transnordestina, s/n Novo Horizonte, 44036-900 Feira de Santana, BA, Brazil

## Abstract

*Background*. The Self-Reporting Questionnaire (SRQ-20) is widely used for evaluating common mental disorders. However, few studies have evaluated the SRQ-20 measurements performance in occupational groups. This study aimed to describe manifestation patterns of common mental disorders symptoms among workers populations, by using latent class analysis.* Methods*. Data derived from 9,959 Brazilian workers, obtained from four cross-sectional studies that used similar methodology, among groups of informal workers, teachers, healthcare workers, and urban workers. Common mental disorders were measured by using SRQ-20. Latent class analysis was performed on each database separately.* Results*. Three classes of symptoms were confirmed in the occupational categories investigated. In all studies, class I met better criteria for suspicion of common mental disorders. Class II discriminated workers with intermediate probability of answers to the items belonging to anxiety, sadness, and energy decrease that configure common mental disorders. Class III was composed of subgroups of workers with low probability to respond positively to questions for screening common mental disorders.* Conclusions*. Three patterns of symptoms of common mental disorders were identified in the occupational groups investigated, ranging from distinctive features to low probabilities of occurrence. The SRQ-20 measurements showed stability in capturing nonpsychotic symptoms.

## 1. Introduction

Common mental disorders (CMDs) are a group of nonspecific symptoms, without any clinical classification relating to psychosocial, occupational, and social context factors. The name CMD encompasses nonpsychotic symptoms, characterized by insomnia, fatigue, irritability, forgetfulness, difficulty in concentrating, and somatic complaints that can coexist in the presence of other comorbidities and define a state of emotional transitory vulnerability, with incapacitating characteristics [1].

The global prevalence of common mental disorders was estimated as 17.6% during the last 12-month period [2]. The World Health Organization [3] has proposed the Self-Reporting Questionnaire (SRQ) as a screening instrument for mental disorders in developing countries, in population-based surveys on individuals who use primary healthcare services [4]. SRQ has undergone structure modifications from its initial composition of 30 items to 20 items relating to psychosomatic symptoms [5]. From the 1980s until today, the validity of SRQ-20 measurements has been evaluated in specific population groups and among healthcare service users [3, 4, 6–10]. However, few studies have evaluated the performance of SRQ-20 measurements in occupational groups [11, 12].

Despite the small number of validation studies about SRQ-20 measurements among workers, this questionnaire has been frequently used in the screening for mental disorders in the labor force [13–18]. The interpretation of the SRQ-20 score is hampered by the wide variation in the cut-off points for diagnostic suspicion, cultural differences, and the diverse work contexts that hinder the analysis of the patterns of symptoms among different occupational groups [3, 19, 20].

Studies [17, 18] have associated common mental disorders with work process characteristics like instability and job dissatisfaction, low wages, earning by productivity, hard work, and job supervision. These conditions trigger a set of signs and symptoms that mostly correspond to CMD and, regardless of the activity performed, reveal a sense of sadness, reduced ability to enjoy daily activities, and decreased concentration and decision-making [21].

Evaluation of measurements used for screening for CMDs in occupational contexts represents a methodological advance for psychiatric epidemiology. The current concept of validity requires the construction of a structured argument and the production of evidence for supporting or refuting interpretations suggested by the scores of a specific instrument [22]. Even with the technical and methodological advances in the analyses, limitations on the screening instruments for evaluations of the main dimensions that compose common mental disorders still remain.

This study aimed to describe manifestation patterns of common mental disorders symptoms among workers populations, by using latent class analysis.

## 2. Methods

This study involves four cross-sectional design surveys conducted on populations of workers that were selected through specific sampling procedures.

*Study 1: Informal Workers*. This epidemiological survey used systematic sampling to select 1,458 open market traders, street dealers, and motorcycle taxi drivers in Feira de Santana, Bahia, in 2008.

*Study 2: Teachers*. This survey is a census on 4,496 teachers at the 365 kindergarten and elementary schools of the public network of Salvador, Bahia, in 2006 [13].

*Study 3: Healthcare Workers*. This survey was a multicenter study with primary healthcare workers from four municipalities from the State of Bahia (Feira de Santana, Jequié, Santo Antônio de Jesus, and a health district in Salvador). A proportional random stratified sample has selected 2,448 workers, from 2012 to 2013.

*Study 4: Urban Workers*. This survey was a random sample of 1,557 individuals, representing workers over 15 years of age, stratified according to subdistricts of the urban zone of Feira de Santana City, in 2007.

In all four studies, common mental disorders were evaluated using the Self-Reporting Questionnaire (SRQ-20).

Latent class analysis was used. This method underlies a wide analytical spectrum based on structural equation models, and it is used when evaluation of a measurement and classification model based on a group of answers and exploration of possible associations is desired. Latent class models refer to situations in which the variable and its indicator are categories [23]. The latent class model is often considered to be analogous to factor analysis for categorical data, because of the possibility of data reduction. However, factor analysis takes the structure of the variables and their correlations into consideration, while latent class analysis evaluates the structure of the cases through the latent taxonomic structure, which therefore relates it to cluster analysis [3, 23, 24].

The traditional latent class model has limited psychometric analyses on instruments that evaluate multidimensional events such as common mental disorders, given that this model does not allow explicit distinction of the dimensional structure [25]. Despite not being appropriate for evaluating the dimensional structure, this technique has become indicated in subjective component descriptions because it is used to analyze representations of multiple variables simultaneously [26].

Latent class analysis was performed on each database separately, following the theoretical assumption that the mental disorders would conform to four dimensions [8, 12]. Initially, extraction of four latent classes was requested. To evaluate the adequacy of the number of classes extracted from the CMD cases gathered, the Vuong-Lo-Mendell-Rubin test, adjusted Lo Mendell Rubin (LRT) test, and parametric bootstrap likelihood ratio test were used. These tests compared a model with *K* classes and a model with (*K* − 1) classes. In the present study, the criterion of concordance of the three tests was used for acceptance of the number of classes, and *p* values < 0.05 were taken to be statistically significant.

Latent class analysis is based on the assumption of local independence, which assumes that, in the latent class model, the variables manifested are independent of one another within the latent classes. For this reason, the local dependence of the items in each database was evaluated. The items were combined in pairs and Pearson's chi-square (*χ* ^2^) and residual *z*-score were calculatedfor all the data in the adjustment model (for all possible pair combinations). When over 50% of the *z*-scores were over 1.96 or below −1.96 and when Pearson's chi-square value was over 50,000, local dependence was considered to be present. To adjust the local dependence, the item fusion method was used [27].

Item fusion was used in accordance with clinical judgment. Thus, in studies 1 and 2, out of the 20 items of SRQ-20, 12 items with local dependence were evaluated and were fused into single items: Q7 (“poor digestion”) and Q19 (“stomach problems”) were named “somatization/digestive”; Q8 (“not thinking clearly”) and Q12 (“difficulty in decision-making”) were considered to be “unsafe behavior”; Q9 (“unhappy”) and Q10 (“crying more than normally”) were classified as “unhappy.” Items Q11 (“not enjoying activities”) and Q13 (“work suffering”) were grouped under “work suffering”; Q14 (“not feeling life is useful”) and Q16 (“feeling worthless”) were considered to be “feeling useless”; Q18 (“always feeling tired”) and Q20 (“easily tired”) received the name “tiredness.”

In study 3, out of the 20 items evaluated, eight presented local dependence: Q7/Q19, Q8/Q12, Q9/Q10, and Q18/Q20. In study 4, conditional dependence was found for six items of SRQ-20: Q9/Q10, Q14/Q16, and Q18/Q20. In both of these studies, the same names as described in relation to studies 1 and 2 were used.

Entropy measurements were then evaluated to indicate class separation quality. Values over 0.80 were considered to be excellent for class discrimination [28].

After the quantity and quality of the classes extracted had been analyzed using the methods mentioned above, the classes were evaluated descriptively according to the numbers and proportions of workers participating in each class. The conditional probabilities that items would belong in each class were evaluated, and the affirmative responses for each item forming part of the questionnaire were taken into consideration. Since this method allows items to simultaneously belong to more than one class, the subgroups forming the CMD construct were judged based on items presenting higher conditional probability in the preestablished classes.

Finally, the conditional probabilities were presented in charts. The *x*-axis presented the questionnaire items and the *y*-axis presented the probability of answering “yes” to a certain item, considering that it belonged to a certain class. For this analysis, the MPLUS software, version 7, was used [29].

The reliability of the latent classes was evaluated by means of the latent class reliability coefficient (LCRC) test, as estimated using the analysis method of Mokken's scale [30, 31]. This scaling procedure is indicated for dichotomous and/or polytomous items. Mokken's estimate, which is a group of reliability statistics comprising Molenaar Sijtsma (MS) statistics, Guttman's lambda 2, and the latent class reliability coefficient (LCRC), was calculated using the R software of the Foundation for Statistical Computing.

The four studies mentioned in our paper were approved by Ethical Committees, before they have been carried out. All participants involved in the four studies signed the consent form for participation. The present study was also approved by an Ethical Committee (CAAE 18723813.9.0000.5030).

## 3. Results

The informal workers evaluated in study 1 were characterized by low education level (95.9%), with equal distribution according to sex and predominance of the age groups < 30 years and 30–45 years. Study 2, which evaluated teachers, involved a predominantly female population (92.0%) aged 30–45 years, with high prevalence of technical/tertiary education level (82.1%). The healthcare workers of study 3 were mostly female (80.6%), aged 30–45 years (44.7%), and the largest proportions had elementary and technical/tertiary education levels (42.9% and 41.3%, resp.). The urban workers evaluated in study 4 presented a higher percentage of females (54.7%), with predominance of the age groups < 30 years and 30–45 years; 55.9% had technical/tertiary education level (Table 1).

The latent class analysis followed the established statistical criteria for acceptance of the number of classes extracted and revealed that three classes had been extracted for all the studies. The entropy values used for evaluating class separation reached values that were either high (from 0.78 to 0.80) or close to the reference value for excellence (higher than 0.80).

Estimators evaluated using Mokken's method presented acceptable reliability values for the number of classes extracted in the latent class model, in all the studies. The latent class reliability coefficient (LCRC) was used as a parameter in this analysis. Studies 1 and 3 presented higher reliability indicators: 0.93 and 0.91, respectively. The lowest value was found in study 4: 0.84 (Table 2).

The worker distribution into classes, in the four studies, followed the same pattern: the lowest proportion in class I, followed in increasing proportions by classes 2 and 3.

**Figure 1:**
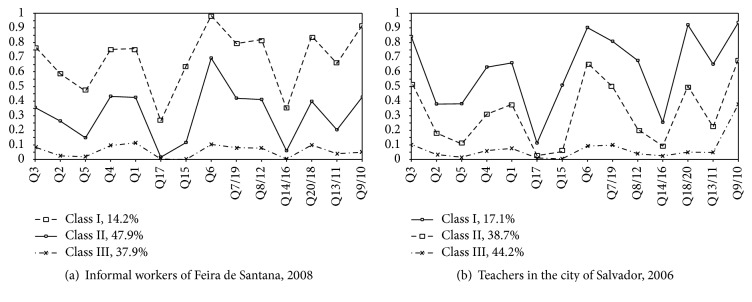
Conditional probabilities of positive responses to SRQ-20 items according to latent class analysis between informal workers and teachers. Q3: sleeping problems; Q2: lack of appetite; Q5: shaking hands; Q4: being frightened; Q1: headaches; Q17: thinking of ending life; Q15: loss of interest in life; Q6: feeling nervous; Q7/19: “somatization/digestive”; Q8/12: “unsafe behavior”; Q14/16: “feeling useless”; Q18/20: “tiredness”; Q13/11: work suffering; Q9/10: “unhappy.”

**Figure 2:**
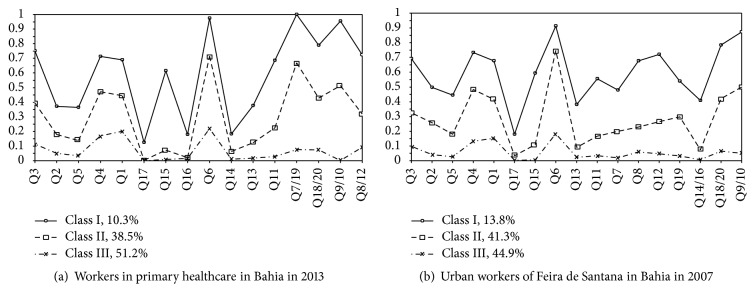
Conditional probability of positive responses to SRQ-20 items according to latent class analysis between healthcare workers and urban workers. Q3: sleeping problems; Q2: lack of appetite; Q5: shaking hands; Q4: being frightened; Q1: headaches; Q17: thinking of ending life; Q15: loss of interest in life; Q6: feeling nervous; Q7: Is your digestion poor?; Q19: stomach problems; Q7/19: “somatization/digestive”; Q8: Do you have trouble thinking clearly?; Q12: difficulty in decision-making; Q8/12: “unsafe behavior”; Q14: not feeling life is useful; Q16: feeling worthless; Q14/16: “feeling useless”; Q18/20: “tiredness”; Q13: Is your daily work suffering?; Q11: not enjoying activities; Q9/10: “unhappy.”

In all the studies, class I concentrated on the workers with highest probability of positive responses to the items in SRQ-20 and revealed better criteria for suspecting common mental disorders. In particular, there were higher probabilities for item Q6 (“Do you feel nervous, tense or preoccupied?”) and for the combined items Q18/20 (“feeling tired”) and Q9/10 (“sadness”), which discriminated this class well (Figures 1 and 2).

Among the professional categories evaluated, study 2 (teachers) presented a higher proportion of workers grouped in class I (17.1%), followed by the informal workers of study 1 (13.4%). Healthcare workers were the category with the lowest percentage of workers in this class (10.3%).

Class II presented a positive response profile similar to what was found for class I, thus discriminating workers with intermediate levels of probability for responses to items belonging to the components of anxiety, sadness, and decreased energy that shape CMDs. Studies 1 and 4 presented higher proportion of workers in this class: 47.9% and 41.3%, respectively.

Class III was composed of subgroups of workers with low probability (under 10%) of responding positively to questions screening for CMDs. Healthcare workers (study 3) were the category with the highest proportion of workers in this class (51.2%) (Figure 2(a)).

In all the studies, Q17 (“Have you thought of ending your life?”) was the item presenting least probability of a positive response among the classes extracted. The same pattern of low probability of an affirmative response was followed by item Q15 (“Have you lost interest in things?”) (Figures 1 and 2).

## 4. Discussion

The latent class model did not allow isolation of items in specific dimensions. Instead, it revealed conditional positive response patterns associated with the extracted classes, thereby allowing discrimination of subgroups that had not been directly observable in the participating studies. The profiles showed variation in intensity, that is, high, intermediate, and low probabilities for positive responses to the questionnaire. Thus, it was possible to identify traits of the anxious/depressive components, represented by higher probabilities of positive responses given by the workers in relation to items Q6, Q9/10, and Q18/20.

The method has been used in healthcare research, especially in the field of psychiatry. The classes translate phenotypes of clinical and/or behavioral manifestations. However, difficulties in identifying the number of classes that best represents the phenomenon evaluated remain [24]. Despite the limitations imposed by the latent class method and cross-sectional study, the CMD manifestation patterns were similar among the professional categories evaluated.

Class III composite with workers with low probability of positive responses to SRQ-20 items was significant in all studies. This result showed the work context of the evaluated groups and reflects positive interference of autonomy in the workplace [17, 20].

The latent class reliability (LCR) estimator confirmed that the number of classes extracted represented the investigated populations, thus satisfactorily capturing the latent features composing CMDs in the different groups evaluated. It is postulated that the method applied in the present study is less skewed in evaluating real reliability than the methods of Guttman's lambda 2, Molenaar Sijtsma (MS), and split-half reliability coefficient [32, 33]. The method of class reliability estimation allowed a more accurate analysis, because the terms were estimated with lower restriction, thus allowing evaluation of multidimensional instruments through considering the essential effects of tau-equivalence and double monotonicity.

The CMD evaluation in multidimensional categories incorporated advances in investigations on mental illness of occupational scope. The screening to obtain homogeneous groups for research and the action strategies established for maintaining mental health were methodological advances in this field. However, there is no consensus on the most appropriate type of multivariate analysis for multidimensional model evaluation, given that any statistical model translates a simplified picture of reality [1]. Scores from instruments evaluating subjective content will reflect the underlying construct with higher or lower precision, but never perfectly. Therefore, validity is considered to be an inferred property from measurements produced by the instrument and needs to be established for each intended evaluation context [22].

The CMDs screened by SRQ-20 reflect transient symptoms and capture recent changes to consider the work environment [33]. However, the persistence of symptoms denotes depressive behavior profiles and anxious somatoform scope and is associated with high burden of disability, missed work, and comorbidities among workers.

## 5. Conclusions

Three patterns of symptoms of common mental disorders were identified in the occupational groups investigated, ranging from distinctive features to low probabilities of occurrence. The SRQ-20 measurements showed stability in capturing nonpsychotic symptoms. Although most workers present low probability of presenting common mental disorders, the symptom patterns encompassing sadness, anxiety, and energy expenditure were very frequent in all occupational groups.

## Figures and Tables

**Table 1:** Sociodemographic characteristics of the populations of the four studies.

Study**:** population (*N*)	*n*	%
*Study 1: informal workers (1,458)*		
Sex		
Female	728	49.9
Age group		
<30 years	537	36.8
30 to 45 years	553	37.9
>45 years	368	25.2
Education level (1,438)		
Elementary	1,379	95.9
Technical/tertiary	9	0.6
Without qualification	50	3.5

*Study 2: teachers (4,496)*		
Sex (4,342)		
Female	3,994	92.0
Age group (4,302)		
<30 years	773	18.0
30 to 45 years	2,289	53.2
>45 years	1,240	28.8
Education level (4,398)		
Elementary	717	16.3
Technical/tertiary	3,609	82.1
Postgraduate	72	1.6

*Study 3: healthcare workers (2,448)*		
Sex (2,421)		
Female	1,951	80.6
Age group (2,395)		
<30 years	581	24.3
30 to 45 years	1,071	44.7
>45 years	743	31.0
Education level (2,419)		
Elementary	1,038	42.9
Technical/tertiary	1,000	41.3
Postgraduate	381	15.8

*Study 4: urban workers (1,557)*		
Sex (1,557)		
Female	851	54.7
Age group (1,557)		
<30 years	576	37.0
30 to 45 years	584	37.5
>45 years	397	25.5
Education level (1,269)		
Elementary	536	42.3
Technical/tertiary	710	55.9
Without qualification	23	1.8

**Table 2:** Summary of the latent class analysis in four professional categories.

	Informal workers (*N* = 1,458) 5^*∗∗*^		Teachers (*N* = 4,397) 162^*∗∗*^		Healthcare workers (*N* = 2,448) 36^*∗∗*^		Urban workers (*N* = 1,556) 14^*∗∗*^	
	*n*	%	*n*	%	*n*	%	*n*	%
Class I	200	14.2	724	17.1	324	10.3	215	13.8
Class II	712	47.9	1736	38.7	923	38.5	642	41.3
Class III	546	37.9	1935	44.2	1286	51.2	699	44,9
Entropy	0.78		0.72		0.80		0.76	
VLMR^a^ test^*∗*^	0.00		0.00		0.00		0.00	
LMR-LRT^b^ test^*∗*^	0.00		0.00		0.00		000	
PB^c^ test^*∗*^	0.00		0.00		0.00		0.00	

Latent class reliability estimators								
Molenaar Sijtsma	1.0		0.86		1.0		0.85	
Lambda	0.93		0.86		0.92		0.85	
LCRC^d^	0.93		0.86		0.91		0,84	

^*∗*^ *p* value.

^*∗∗*^Number of imputed pattern observations.

^a^VLMR: Vuong-Lo-Mendell-Rubin.

^b^LMR-LRT: adjusted Lo Mendell Rubin (LRT) test.

^c^PB: parametric bootstrapped maximum likelihood.

^d^Latent class reliability coefficient.
